# Evaluating the Impact of Age, Acoustic Exposure, and Electrical Stimulation on Binaural Sensitivity in Adult Bilateral Cochlear Implant Patients

**DOI:** 10.3390/brainsci10060406

**Published:** 2020-06-26

**Authors:** Tanvi Thakkar, Sean R. Anderson, Alan Kan, Ruth Y. Litovsky

**Affiliations:** 1 Waisman Center, University of Wisconsin-Madison, Madison, WI 53705, USA; tanvi.thakkar@wisc.edu (T.T.); sean.anderson@wisc.edu (S.R.A.); 2 School of Engineering, Macquarie University, Sydney, NSW 2109, Australia; alan.kan@mq.edu.au

**Keywords:** hearing loss, binaural sensitivity, cochlear implant

## Abstract

Deafness in both ears is highly disruptive to communication in everyday listening situations. Many individuals with profound deafness receive bilateral cochlear implants (CIs) to gain access to spatial cues used in localization and speech understanding in noise. However, the benefit of bilateral CIs, in particular sensitivity to interaural time and level differences (ITD and ILDs), varies among patients. We measured binaural sensitivity in 46 adult bilateral CI patients to explore the relationship between binaural sensitivity and three classes of patient-related factors: age, acoustic exposure, and electric hearing experience. Results show that ILD sensitivity increased with shorter years of acoustic exposure, younger age at testing, or an interaction between these factors, moderated by the duration of bilateral hearing impairment. ITD sensitivity was impacted by a moderating effect between years of bilateral hearing impairment and CI experience. When age at onset of deafness was treated as two categories (<18 vs. >18 years of age), there was no clear effect for ILD sensitivity, but some differences were observed for ITD sensitivity. Our findings imply that maximal binaural sensitivity is obtained by listeners with a shorter bilateral hearing impairment, a longer duration of CI experience, and potentially a younger age at testing. 198/200.

## 1. Introduction

Normal hearing (NH) listeners develop sensitivity to acoustic differences between the ears at a very young age. This allows listeners to unmask speech from noise and localize sound sources [1]. In order to accurately localize sounds on the horizontal plane, the binaural hearing system relies on computing the interaural time difference (ITD) and interaural level difference (ILD) from the incoming acoustic signal. Improving binaural hearing has become highly relevant in the clinical domain due to the appreciation of deaf patients’ needs to communicate and function in realistic, complex auditory environments. It is well-established that the benefits of cochlear implantation in one ear include sound awareness, a better quality of life, and significant improvements in speech perception [2,3,4]. The standard of care for deaf individuals has changed in the past two decades to providing bilateral, rather than unilateral, cochlear implants (CIs). However, bilateral implantation does not guarantee access to the acoustic cues that facilitate the beneficial use of the binaural hearing system (i.e., spatial unmasking and sound source localization). When patients with bilateral CIs wear their clinical processors, they typically have limited access to ITDs and ILDs, resulting in less accurate sound source localization [5,6,7].

Studies to date have shown that patients with bilateral CIs primarily use ILDs for sound localization because clinical processors can provide usable ILDs to some extent [6,7,8]. In contrast, ITD cues are poorly conveyed through clinical processors, leading to a gap in performance between CI patients and NH listeners in sound localization tasks [5,7,9,10]. In a research setting, researchers can fit CI patients with specialized bilateral research processors (see [11] for review) that synchronize the timing of electrical stimulation between the two ears. When listening through research devices, bilateral CI patients show some sensitivity to ITDs presented at low rates of stimulation, typically around 100 pulses per second [10,12]. The underlying factors that contribute to the limited ITD sensitivity observed in patients with bilateral CIs are still poorly understood, though a range of factors has been reviewed in prior literature [13,14]. These factors include: (1) poor neural survival in one or both ears, resulting in neural asymmetries between the ears, (2) surgical limitations due to asymmetrical placement of electrodes in the two ears, (3) lack of synchronization between clinical speech processors in the two ears, (4) reduced preservation of binaural cues due to the signal processing and removal of temporal fine structure, (5) use of high-rate pulsatile stimulation in most CI clinical processors, and (6) underlying etiological factors [10,11,13]. Most studies have explored only one or two of these factors in isolation.

The present study investigated the relationship between several etiological and experiential factors and sensitivity to ILDs and ITDs in adult bilateral CI patients. These factors were categorized as (1) age-related factors (i.e., age at onset of deafness and age at testing); (2) acoustic experience/exposure factors (i.e., total years of acoustic exposure and years of bilateral acoustic impairment); (3) experience with electric hearing (i.e., years of experience with a CI and years of bilateral CI experience). The goal of the present study was to uncover how these factors concurrently contribute to ILD and ITD sensitivity.

### 1.1. Age-Related Factors

Two age-related factors have demonstrated reliable relationships with binaural sensitivity and were investigated in the present study: the age at onset of deafness and the age at experimental testing. An earlier age of onset of deafness has been associated with poorer binaural hearing performance, specifically ITD sensitivity. Prior studies that examined the relationship between age at onset of deafness and binaural sensitivity in bilateral CI users have found an association between ITD sensitivity and age at onset of deafness between adult, late-onset (>18 years), mid-late childhood onset (between 18- and 5-years-old), and early onset (<5-years-old) groups [10,15]. Patients that had an onset of deafness less than five years of age showed no measurable sensitivity to ITDs, while all patients, regardless of having a late or early age at the onset of deafness, demonstrated ILD sensitivity. This finding suggests that exposure to acoustic hearing at a young age is important for attaining sensitivity to ITDs but not necessarily ILDs.

The relationship between binaural sensitivity and age at onset of deafness is also supported by animal studies. Recent work in rabbits has found that the response of neurons in the inferior colliculus (IC) to ITDs presented through electrical pulse trains were more likely to show sustained, excitatory responses and less likely to show suppression in early-deafened rabbits than adult-deafened rabbits. In addition, early-deafened rabbits had reduced overall firing in ITD-sensitive neurons compared to adult-deafened animals. This work implies that sensitivity to ITDs of neurons involved in binaural processing in adult animals is significantly reduced following an early onset of deafness, thus leading to a sub-optimal encoding of ITDs [16].

Aging during adulthood can also play a role in binaural sensitivity, as seen in human studies conducted with older adults. Both behavioral and electrophysiological measures have shown that aging leads to declines in the ability to process binaural cues including ITDs and interaural phase differences (IPDs) in individuals with normal audiometric thresholds [17,18]. Behavioral measures with older listeners have shown that ITD thresholds measured using a 100-Hz acoustic pulse train are typically double that of younger listeners, particularly at very low sensation levels [19,20,21]. While poorer ITD sensitivity appears to be associated with increasing age, most existing evidence suggests that there is no age effect on ILD processing [18,20,22,23].

Electrophysiological measures have been used in prior studies to understand the physiological correlates of sensitivity to interaural differences in older and younger listeners, specifically for ITDs. For example, cortical responses to ITDs differ in older (mean = 70.0 years) versus younger (mean = 24.9 years) listeners [17]. In older adults, larger cortical auditory evoked potential magnitudes are measured in response to static ITDs compared to younger listeners. Conversely, significantly longer latencies with smaller magnitudes for the first negative peak (N1) and second positive peak (P2) are observed for older versus younger listeners when a dynamically changing ITD is presented to listeners, suggesting poorer neural synchrony [17,24]. Additional work has found that the ability to use interaural temporal information to aid in the release from masking (e.g., binaural masking level differences; BMLDs) also declines with increasing age [19]. BMLDs are measured as the difference in the threshold of the signal (in dB) when the signal and masker have the same interaural phase and level relationships in the two ears compared to when the interaural phase and/or level of the signal and masker are of opposite or different phase. In practice, this means that a signal is easier to detect when located in a different position relative to another source. Results from these studies suggest that the ability to use ITDs to localize sound sources and binaurally unmask sounds is negatively impacted by older age.

### 1.2. Acoustic and Impaired Experience/Exposure Factors

The degree of acoustic exposure and bilateral impairment can also contribute to binaural sensitivity. Thus, we investigated the total years of acoustic exposure and years of bilateral hearing impairment in this study. The best outcome attainable by patients might be limited if they experienced long periods of auditory deprivation. Prior work has shown that patients who are fitted with CIs and tested on speech understanding tasks between two post-operative visits have demonstrated that, with longer durations of severe-to-profound hearing loss before implantation, they show less improvement in behavioral tasks than in patients with a shorter duration [25].

Studies with single-neuron recordings in non-human species provide insight into the role of acoustic exposure on binaural sensitivity. In acutely deafened animals, the encoding of neurally evoked ITDs presented through electrical stimulation of a CI can provide just as accurate encoding of ITDs as those presented with acoustic stimulation [26]. This finding suggests that the length of acoustic impairment might be a key factor contributing to poor vs. good sensitivity to ITDs and not the mode of stimulation. In support of this conclusion, ITD tuning of neurons can be negatively impacted by early-deafened animals with prolonged periods of no acoustic exposure compared with adult-deafened animals [16]. Together, these findings are consistent with work done in humans using psychophysical approaches, suggesting that deprivation of acoustic stimulation before CI stimulation can result in poorer ITD sensitivity.

Another factor for consideration is the degree of relative impairment in each ear, either neurally or behaviorally, that can be affected by prolonged periods of monaural input when one ear receives significantly more stimulation than the other ear [27,28,29]. Asymmetry in hearing impairment can fall along a spectrum ranging from complete loss of hearing in one ear (unilateral deafness) to complete loss of hearing in both ears (bilateral deafness). Prior work has demonstrated that the degree of asymmetry in acoustic impairment, or unilateral versus bilateral acoustic impairment, can play a role in ITD sensitivity. Cats with bilateral deafness can exhibit overall reduced responsiveness in the auditory cortex, whereas in unilaterally deafened cats, cortical responsiveness was found to increase when stimulation was presented to the contralateral side, a sign of aural dominance. Most importantly, sensitivity to ITDs was reduced in unilaterally compared to bilaterally deafened cats. Complete unilateral impairment can create greater cortical asymmetries, resulting in a greater loss of binaural functionality [30,31].

The impact of bilateral hearing impairment on binaural sensitivity has also been investigated in children that use hearing aids. One study investigated whether bilateral hearing impairment in children with hearing aids impacted binaural processing. They found that ITD perception is abnormal in children that used bilateral hearing aids, but that ILD perception was similar to the NH control group [32]. This study highlights how impaired bilateral input in bilaterally implanted or bimodal individuals can impact how a listener benefits from binaural processing (for ITDs but not for ILDs). However, it is unknown whether bilateral hearing impairment impacts adult bilateral CI listeners in the same way.

In summary, these studies indicate that prolonged periods with no auditory stimulation or bilateral hearing impairment prior to CI stimulation can lead to poorer processing of ITDs. Because it is difficult to determine the degree of asymmetry in patients, in this study, we will characterize the effect of patients’ impairment on binaural functionality using the total years of bilateral hearing impairment.

### 1.3. Experience with Electric Hearing

It is still unclear how the length of experience with electric hearing directly influences binaural sensitivity in patients with bilateral CIs. As there are limited data on the relationship between experience with electric hearing and binaural sensitivity, research conducted in children may provide insight into the influence of electric hearing.

Gordon and colleagues have conducted much of the work on this topic in children. Overall, the perception of ILDs develops early in life in children with bilateral CIs [33,34,35]. When ILDs are presented to NH children and to age-matched bilateral CI children, the cortical representation of ILDs in bilateral CI children is not well developed compared to NH children [36]. However, the behavioral data show that bilateral CI listeners are still able to lateralize ILDs reliably well, suggesting that having electric hearing in both ears might negatively impact ILD cortical processing but still retain behavioral ILD perception. Another study showed that when comparing children with long-term unilateral CI experience, short-term unilateral CI experience, and simultaneously implanted long-term bilateral CI experience, all groups of children were able to discriminate left vs. right using ILDs, even in the group that had experienced hearing from an implant in one ear for most of their lives. Further, the group with longer-term bilateral CI use performed as well as their normal-hearing peers [35]. Supporting the possibility of a similar performance between children with CIs and NH, data from [37] showed that behavioral ILD sensitivity in bilateral CI children is within the range of performance for NH children, regardless of whether those children were implanted sequentially or simultaneously. Hence, ILD perception appears robust regardless of whether the experience was unilateral, bilateral, or long-term bilateral [33,35].

In contrast to ILDs, longer experience with bilateral CI use is needed for ITD sensitivity to develop [33,35]. Using cortical response measures, studies have shown that children who are deaf from a young age and simultaneously-implanted in both ears still do not fully overcome the effects of bilateral deafness. Specifically, cortical responses to ITDs in NH children typically show reduced activity in the auditory cortex ipsilateral to the leading ITD, as well as a unique weighting in the left auditory cortex. This has not been observed in children with bilateral CIs, suggesting that the cortical response to ITDs is reduced compared to NH listeners [38]. Further, Gordon et al. [35] showed that, behaviorally, children with longer bilateral CI use were the only group capable of detecting ITDs, suggesting that the perception of ITDs was promoted by longer bilateral CI use. The ability to detect these cues was not observed in the group that was older and had long-term unilateral use, nor was this effect observed in the younger children with short-term unilateral experience (approximately 2.2 years of bilateral experience, on average). Hence, behavioral response to ILDs may not be dependent on CI experience, but the behavioral response to ITDs might be partially recovered with longer bilateral CI use.

### 1.4. Understanding Binaural Sensitivity in Bilateral Cochlear Implant Users

**Figure 1:**
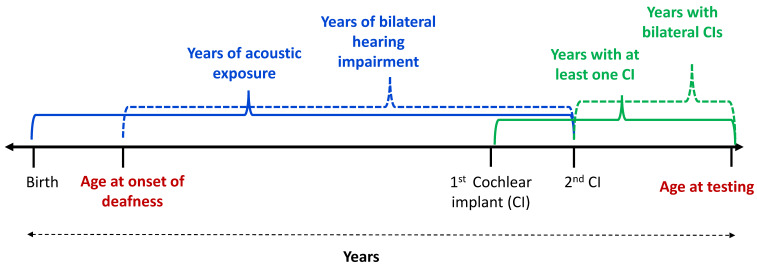
A schematic depicting the calculation for each factor investigated.

While typical candidates for cochlear implantation have severe-to-profound sensorineural hearing loss [36], the history of hearing can vary greatly for the time between the onset of hearing loss to when a CI is provided. Behavioral and physiological experiments have taught us that factors like age, acoustic exposure, and experience with electric hearing play an important, yet complex role in binaural sensitivity outcomes. At present, it is not clear how each of these factors might interact with one another, as they are likely to have complex relationships with binaural sensitivity. Previous studies have only investigated one of these factors at a time. Interpreting the contributions of etiological factors on binaural sensitivity in adult CI patients is often difficult because of the large inter-patient variability and smaller sample sizes [13,14]. Here, we present the first set of results from a larger population of bilateral CI participants, having a wide range of etiological differences. The goals of the current study were to determine which of these etiological differences provide the most robust predictions of binaural sensitivity. It remains to be determined whether age, bilateral deafness, or CI experience play a synergistic role in binaural sensitivity. The second goal of the current study was to explore the potential interaction between these factors. Finally, most studies focus on either ILD or ITD sensitivity alone. While aging, acoustic exposure, and experience with electric hearing show a more robust effect on ITD sensitivity, another goal was to determine whether ILD and ITD sensitivity were related to one another across listeners. Based on prior findings, it was hypothesized that sensitivity to ILDs would be robust, regardless of age, hearing exposure, or CI experience, while sensitivity to ITDs would be demonstrated in listeners with a longer history of acoustic hearing exposure and younger versus older age at onset of deafness. The factors we investigated and their calculation are described in Figure 1.

## 2. Materials and Methods

### 2.1. Listeners

Forty-six adult bilateral CI listeners with at least one year of experience with bilateral CI hearing participated in this study. All 46 listeners participated in an ITD discrimination experiment, while 31 of the listeners participated in an ILD discrimination experiment. Listeners traveled to the University of Wisconsin–Madison for three days of testing and were paid a stipend for their participation. Listener etiology is displayed in Table 1. All listeners had Cochlear Ltd. (Sydney, Australia) implants (CI24 and CI512 family of implants). These devices have 24-electrodes (22 intracochlear and two ground electrodes), with inter-electrode spacing ranging from 0.4 mm at the apical end to 0.8 mm at the basal end. Electrodes are numbered such that 22 is the apical-most electrode and 1 is the basal-most electrode. One listener (IBR) was unable to complete testing with one of the electrode sites in the ITD discrimination experiment. Experimental procedures followed the regulations set by the National Institutes of Health and best practice guidelines as outlined in [11]. The experiments were approved by the University of Wisconsin’s Health Sciences Institutional Review Board with the approval code 2015-1438.

### 2.2. Equipment and Stimuli

Stimuli were delivered via specialized bilateral research processors. Thus, the listeners’ external processors were removed and replaced with bilaterally-synchronized L34 research processors or RF Generator XS (Cochlear, Ltd., Sydney, Australia). The research processors were controlled using MATLAB (Mathworks, Natick, MA) via the Nucleus Implant Communicator (NIC2 for L34s, NIC3 for RF Generator). This setup has been used extensively by the authors for psychophysical experiments with bilateral CI patients (see [39,40,41,42] for details). A personal computer with custom-made software in MATLAB was used to deliver stimuli and collect data. Responses were made on a touchscreen connected to the computer. Stimuli were anodic-phase leading, biphasic pulse trains presented in monopolar (MP1 + 2) configuration. All stimuli were 300 ms in duration, had constant amplitude, and were presented at a rate of 100 pulses-per-second (pps). No onset or offset ramps were added to the stimulus. The phase duration of each electrical pulse was set according to the patient’s clinical MAP. This was typically a 25-µs phase duration with an 8-µs phase gap.

### 2.3. Mapping

The loudness level for all stimuli was determined through a mapping procedure in which the experimenter adjusted the level of the stimulus in clinical current units (CUs) for all even-numbered electrodes. (For Cochlear-brand devices, clinical CUs range from 0 to 255 and represent logarithmically stepped stimulation current levels.) The loudness map defines the range of current units that generate an audible percept between threshold (T) and most comfortable (M) loudness levels at each electrode. During the mapping session, listeners reported the perceived loudness of the electrical stimulus. The T-level is the level (in CUs) that the listener can detect before the sound becomes inaudible. The comfortable level, or C-level, is the level that the listener reported to be comfortably loud and was willing to listen to all day. Finally, the M-level is defined as the highest current level that the listener can tolerate without being uncomfortably loud. Levels for odd-numbered electrodes were interpolated from the levels found for the even-numbered electrodes. 

C-levels were adjusted to ensure that the loudness perceived at each electrode, both within and across ears, was equal. C-levels were compared across electrodes within an ear to ensure the same loudness. This was achieved by stimulating the electrodes one at a time with an inter-stimulus interval of 100 ms. Listeners indicated the electrodes that were noticeably louder than others and those C-levels were adjusted. The experimenter adjusted levels to attain equal loudness across all electrodes. Across-ear loudness balancing was completed after the pitch matching procedure (described in the next paragraph). A single pitch-matched pair was played simultaneously, varying the C-level across ears, until the listener perceived the sound as equally loud and coming from the center of the head.

### 2.4. Pitch Matching

Three bilateral, pitch-matched electrode pairs along the length of the electrode array were determined for each listener. Across-ear pitch-matching of electrodes serves as a means of matching place-of-stimulation in the cochlea in each ear. Other methods exist for place matching, including finding the interaural electrode pair that yields the “best ITD” sensitivity using psychophysical testing and measurement of the binaural interaction component in the brainstem [43,44]. Although pitch-matching can be influenced by conditions tested and the task parameters [45], our approach usually leads to an interaural pair of electrodes that has relatively good ITD sensitivity in adults [41,44,46].

Two tasks were used to determine pitch-matched pairs: a pitch magnitude estimation (PME) and a direct pitch comparison (DPC) task. For both tasks, the stimulus was presented at C-level. In PME, listeners were asked to rate the pitch of the stimulus on a scale from 1 (low pitch) to 100 (high pitch), while stimulation was presented to a single electrode in either the left or right ear. Listeners were first familiarized with the range of pitch that they are likely to perceive by stimulating each electrode individually. The results of PME were used to determine the range of electrodes that should be used for the DPC task. In the DPC task, a reference electrode in one ear was compared against a subset of electrodes in the other ear using a two-interval, five-alternative forced-choice task. On each trial, the reference electrode was played first, followed by one of the electrodes in the other ear, chosen from the small subset identified from the PME task. The listener’s task was to judge whether the stimulus in the second interval was: “much higher,” “higher,” “same,” “lower,” or “much lower” in pitch compared to the reference electrode. The three pitch-matched electrode pairs were usually tested within the same block, where each of the three reference electrodes was chosen randomly for each trial. From the DPC task, the pair that yielded the highest number of “same” results was chosen as the pitch-matched pair for further testing (see [10] for further details on methodology). The three pitch-matched pairs found for each listener are shown in Table 1. The pairs were selected to span roughly the entire electrode array at the three different locations: apex, middle, and base.

### 2.5. Tasks

#### 2.5.1. ILD Discrimination

ILD just-noticeable-difference (JND) thresholds were estimated using a two-interval, two-alternative forced-choice paradigm with a 300-ms inter-stimulus interval. In each trial, a bilateral stimulus was presented to one pitched-matched electrode pair. The first interval of a trial had an ILD imposed either in the left- or the right-ward direction. In the second interval, an ILD of the same magnitude was applied in the opposing direction to that of the first interval. Subjects responded by indicating the direction of the second interval relative to that of the first interval (i.e., left or right). No training or feedback was provided to the listeners. A method of constant stimuli procedure was used, where ILDs of ±1, ±2, ±5, and ±10 CUs were tested in all listeners. In some instances, additional ILDs were included (such as ±20 CUs) to complete a psychometric function. Each ILD was tested 40 times with 20 left-ward and 20 right-ward ILD trials. The experiment was conducted in blocks, where each block consisted of 10 trials at each ILD (five left-ward and five right-ward). ILDs within each block were tested in random order. The percent correct data were fitted using the “psignift” MATLAB toolbox version 2.5.6 [47]. The ILD JND threshold was estimated from the 70.7% [48] correct point on the psychometric function. It should be noted that the JNDs reported in this paper are approximately 50% smaller than that typically reported using threshold estimation tasks that have a 0-dB ILD in the first interval [49,50].

#### 2.5.2. ITD Discrimination

ITD JND thresholds were estimated using a similar task to the ILD task. ITDs of 100, 200, 400, and 800 μs were tested in all listeners. In some listeners, additional ITDs were included (such as 50, 1600, or 2000 μs) in an attempt to completely cover the range of ITD values where the listener could achieve up to 100% correctness. Each ITD was tested 40 times with 20 left-ward and 20 right-ward ITD trials. The experiment was conducted in blocks, where each block consisted of 10 trials at each ITD (five left-leading and five right-leading). Psychometric functions were fit using the same method in the ILD task. Again, the JNDs reported in this paper are approximately 50% smaller than that typically reported using threshold estimation tasks that have a 0-μs ITD in the first interval [51,52,53,54].

### 2.6. Data Analysis

To investigate the factors listed in Section 1.4 and their ability to predict ITD and ILD JND thresholds, four types of analyses were conducted using three different statistical packages in R (version 3.3.4). 

(1) To understand the effect of place of stimulation, the “lme4” package (version 1.1-13) was used to fit a generalized linear mixed-effects model (GLMM) with a binomial link function for the ILD and ITD JNDs. In this model, “electrode location” (i.e., place-of-stimulation) served as the fixed effect and “subject” served as the random effect.

(2) To explore the impact of etiological factors on JNDs, the “stats” package (version 3.4.4) was used for the linear regression (function: “lm”) and the “MASS” package (version 7.3-49) was used to perform a stepwise model selection (function: “stepAIC”) using the Akaike’s information criterion (AIC). These functions were used concurrently to perform a stepwise multiple regression to determine the effect of the factors mentioned in Section 1.4. The “stepAIC()” function was used to find the best performing model that has the lowest AIC. This was achieved by beginning with a full (i.e., all predictors and their interactions) or null (i.e., only the intercept) model, and specifying the method for stepwise regression (i.e., “forward,” “backward,” or “both”). The method used for this stepwise regression was “both,” or bi-directional, thus predictors were iteratively removed and added from the regression in order to find a resulting model that yielded the lowest overall AIC scores [55]. This function performs an iterative process whereby multicollinearity is addressed by only introducing predictors with the lowest variance inflation factors (VIF). The model with the lowest AIC score was judged to have the best balance between explanatory power and parsimony. As a preprocessing step, Pearson correlations were measured for all combinations of factors described in Section 1.4 using the function “rcorr” from the “Hmisc” package (version 4.2-0). This function can calculate the Pearson pairwise correlations for each pair of variables. VIFs were calculated to estimate how much the variance of a regression coefficient is inflated due to multicollinearity in the model. A VIF of 1 suggests no collinearity of that predictor. For example, a VIF of 1.7 indicates that the variance of a coefficient is 70% greater than if there was no multicollinearity. As a general rule, VIFs greater than 5 should not be used in a multiple regression analysis.

All predictors were mean-centered prior to including them in the model. This simply indicates that the mean of each predictor was subtracted from every value of that predictor. Thus, the zero-point for each predictor is redefined as the mean, but units and their interpretation remain unchanged. Mean centering was completed to combat multicollinearity (linear relationships between combinations of factors) between predictors since many predictors were related to one another (Figure 1 and Table 2).

(3) Categorical analysis was also conducted to further understand the effects of age at onset of deafness because a number of listeners had “unmeasurable,” or “could not determined” JNDs. This analysis allowed us to include both "measurable" and "unmeasurable" JNDs by comparing the number of listeners in two groups with different ages of onset of deafness. 

(4) A linear regression analysis was conducted to explore the relationship between ILD and ITD sensitivity using only “measurable” JNDs.

## 3. Results

### 3.1. Effect of Electrode Pair Location on JNDs

**Figure 2:**
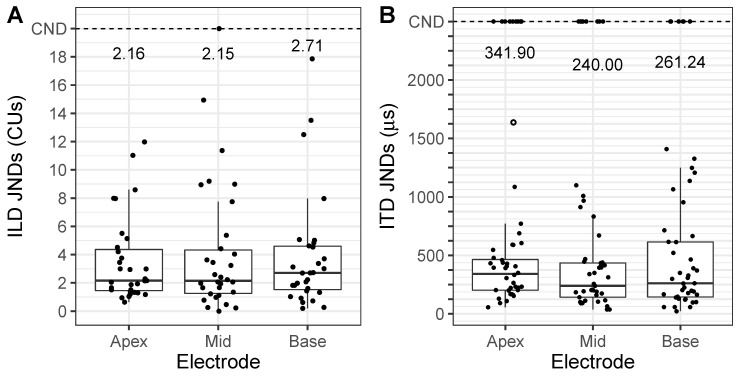
Box plots depicting (**A**) Interaural level difference (ILD) Just-noticeable differences (JNDs) for each electrode pair that was tested. Data shown have all ILD JNDs, including JNDs that could not be determined. Medians represent the data with only JNDs below 20 CUs. Black dashed line indicates the “could not determine” (CND) mark for JNDs > 20 CU. (**B**) Box plots depicting ITD JNDs for each electrode pair that was tested. Data shown have all ITD JNDs, including JNDs that could not be determined. Medians represent the data with only measurable thresholds. Black dashed line indicates the CND mark for JNDs. Subject IBX who was tested with 2000 μs had a JND that exceeded 1600 μs, and therefore the open-circle symbol represents subject IBX.

Figure 2A shows the distribution of ILD JNDs obtained for 31 listeners at each electrode pair location (apex, mid, and base). The median and interquartile range (IQR) for ILD JNDs were 2.16 (IQR = 2.91), 2.15 (IQR = 3.07), and 2.71 (IQR = 3.08) CUs for apex, mid, and base locations, respectively. ILD JNDs greater than the largest ILD tested were removed from the analyses, as they were indicative of poor performance and resulted in an inability to estimate threshold from the psychometric function. These JNDs are labeled as “could not determine” (CND) in Figure 2A. Results from a GLMM analysis found no significant effect of electrode location (F (2, 60.44) = 0.43, *p* = 0.65). Therefore, all measurable ILD JND thresholds were averaged across electrode locations for the remainder of analyses.

Figure 2B shows the ITD JNDs for all listeners at each electrode pair location tested. The median JND for each electrode pair location was 341.90 (IQR = 262.33), 240.00 (IQR = 292.18), and 261.24 (IQR = 470.80) μs for apex, mid, and base locations, respectively. For some electrode locations in some listeners, ITD JNDs could not be measured (because they fell outside of the highest tested ITD; >1600 μs for most subjects) and were removed from the regression analyses. One ITD JND for subject IBX was not removed, as they were also tested with ITD values of 2000 μs and their JND fell below this value (this listener has an open-circle symbol in Figure 2B). JNDs that could not be measured from the psychometric function are labeled as CND in Figure 2B. Results from a GLMM analysis found no significant effect of electrode location (F (2, 68.40) = 1.34, *p* = 0.27). Therefore, all measurable ITD JNDs were also averaged across electrode pair locations in the remaining analyses because of the lack of effect of electrode location.

### 3.2. Effect of Etiological Factors on ILD and ITD JNDs

#### 3.2.1. ILD Just-Noticeable Differences

**Figure 3:**
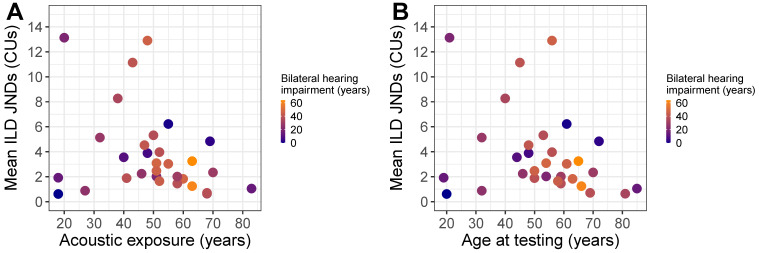
Shows (**A**) ILD Just-noticeable differences (JNDs) as a function of years of acoustic exposure and years of bilateral hearing impairment. Blue-colored symbols indicate fewer years of bilateral hearing impairment and orange-colored symbols indicate greater years of bilateral hearing impairment. (**B**) Shows ILD JNDs as a function of age at testing and years of bilateral hearing impairment. Blue-colored symbols indicate fewer years of bilateral hearing impairment and orange-colored symbols indicate greater years of bilateral hearing impairment.

In order to predict ILD JNDs from the factors listed in Section 1.4, a stepwise multiple regression was conducted. A correlation analysis revealed collinearity between certain factors. These relationships are displayed in Table 2A. The stepwise multiple regression was conducted using all mean-centered predictors. The final model with the lowest AIC (AIC = 71.74, F(4 and 26) = 3.34, *p* < 0.05; $R^{2}\;$= 0.24) is given in Table 3. A significant main effect was found for years of acoustic exposure (*p* < 0.05, VIF = 1.89) but not for years of bilateral hearing impairment (*p* = 0.27, VIF = 1.21) or years with at least one CI (*p* = 0.17, VIF = 1.04). The positive coefficient for years of acoustic exposure indicates that ILD JNDs increased (i.e., ILD sensitivity declined) with more years of acoustic exposure. Additionally, the interaction between years of bilateral hearing impairment and years of acoustic exposure was significant (*p* < 0.05, VIF = 1.72), implying that bilateral hearing impairment moderates the effects of years of acoustic exposure (i.e., a greater number of years of bilateral hearing impairment leads to a smaller effect of acoustic exposure on ILD JNDs). Figure 3A shows ILD JNDs plotted as a function of years of acoustic exposure, color-coded for years with bilateral hearing impairment.

The finding that ILD JNDs increased with more years of acoustic experience indicated by the previous model is counter-intuitive if one assumes that having more access to acoustic hearing is beneficial for performance on psychoacoustical tasks. Based on prior work, we expected that more years of acoustic experience would lead to greater sensitivity to ILDs. Given that we observed a nearly perfect linear relationship between age at testing and years of acoustic exposure, a confound exists between these two variables. Hence, the relationship with ILD sensitivity could be described by either the predictor alone or by their interaction. A secondary stepwise multiple regression was conducted using all predictors except for “years of acoustic exposure” to verify a similar relationship. The final model with the lowest AIC (AIC = 71.22, F(4 and 26) = 3.49, *p* < 0.05; $R^{2}$ = 0.25) is given in Table 4. ILD JNDs were predicted by age at testing (*p* < 0.05, VIF = 1.87) but not years of bilateral hearing impairment (*p* = 0.26, VIF = 1.20) or years with at least one CI (*p* = 0.07, VIF = 1.01). Additionally, there was a significant interaction between years of bilateral hearing impairment and age at testing (*p* < 0.05, VIF = 1.72). Thus, the positive coefficient for age at testing suggests that sensitivity to ILDs decreased as age increased (Figure 3B). This is explored further in the discussion section.

#### 3.2.2. ITD Just-Noticeable Differences

**Figure 4:**
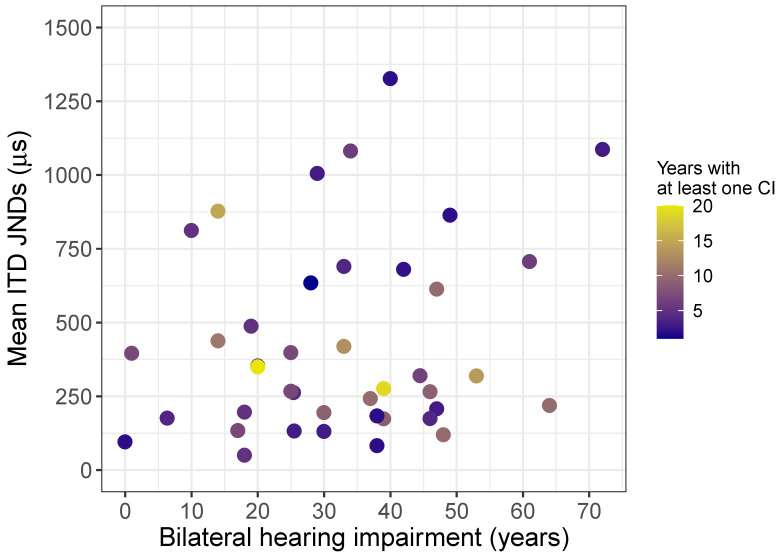
Shows ITD Just noticeable differences (JNDs) as a function of years of bilateral hearing impairment and years with at least one CI. Blue-colored symbols indicate fewer years with at least one CI and yellow-colored symbols indicate greater years of with at least one CI.

In order to predict ITD JNDs from the factors listed in Section 1.4, a stepwise multiple regression was conducted. Due to the different number of participants between the ITD and ILD datasets, relationships of multicollinearity were re-examined (see Table 2B). All mean-centered predictors were included in the step-wise regression. The final model with the lowest AIC (AIC = 471.37) (F(3,37) = 3.23, *p* < 0.05; $R^{2}$ = 0.14) is given in Table 5. No significant main effects for years of bilateral hearing impairment (*p* = 0.25; VIF = 1.01) or years with one CI (*p* = 0.29; VIF = 1.00) were found for the final model. Only the interaction term between years of CI experience and years of bilateral hearing impairment reached significance (*p* < 0.05; VIF = 1.01). Figure 4 shows the mean ITD JNDs as a function of years of bilateral hearing impairment and is color-coded for years with at least one CI. There was a significant, negative interaction between years of bilateral hearing impairment and years with at least one CI. This implies that when years of bilateral hearing impairment and years with at least one CI increase or decrease together, ITD JNDs decrease. Conversely, when years of bilateral hearing impairment and years with at least one CI vary in opposite directions, ITD JNDs tend to increase. This interaction suggests that shorter bilateral hearing impairment counteracts less CI experience, and longer CI experience counteracts longer bilateral hearing impairment.

### 3.3. Categorical Analysis of Age-Related Factors

Prior work suggests that if the developmental time window for the emergence of binaural hearing is interrupted at a young age, the ability to use binaural cues, in particular ITDs, deteriorates in humans [15,34,38]. Age at onset of deafness was not considered in either the final ILD or ITD models because it correlated with other predictors in our dataset. Accordingly, we decided to further investigate the age at onset of deafness separately, based on prior literature. We converted the age at onset to a categorical measure to capture the early and late periods of deafness: childhood (<18-years-old) and adulthood (≥18-years-old). This decision was made because our laboratory previously observed that bilateral CI listeners with mid-late childhood-onset of deafness (<18- and >5-years-old) vary in their ability to demonstrate ITD sensitivity while listeners in the adulthood group (>18 years) all had measurable ITD sensitivity [15]. Another important finding was that many listeners with an age at onset less than 5 years, and between 5 and 18 years had unmeasurable thresholds, specifically for ITD JNDs but not ILD JNDs. Categorizing JNDs allowed all JNDs, whether measurable or unmeasurable, to be included in analyses.

**Figure 5:**
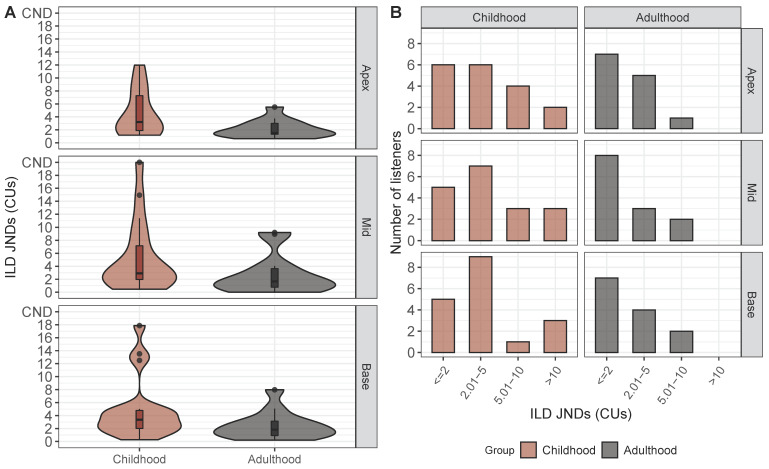
Shows the distribution of (**A**) ILD Just-noticeable differences (JNDs) as a box plot and violin plot for all listeners as a function of age at onset of deafness group. A wider distribution (i.e., greater thickness) on a violin plot corresponds to a greater number of listeners with a given JND on the y-axis. Black dashed line indicates “could not determine” (CND) threshold for ILD JNDs; (**B**) shows histograms of ILD JNDs, for each age group, binned according to levels of performance ranging from “good” (< 2 CUs) to “poor” (>10 CUs).

**Figure 6:**
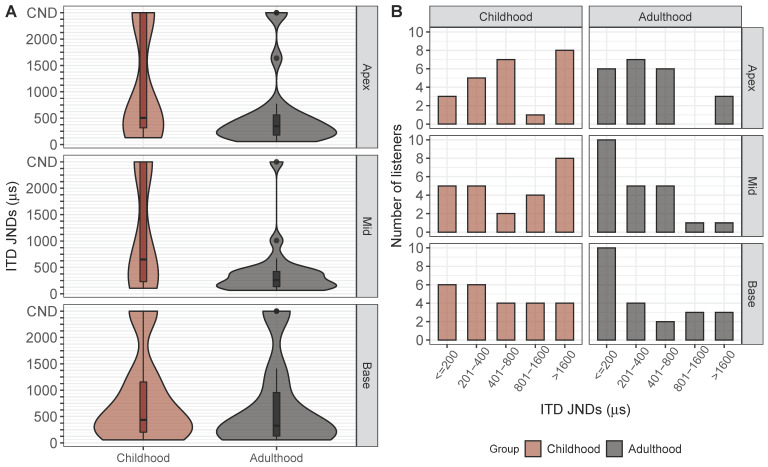
Shows the distribution of (**A**) ITD Just-noticeable differences (JNDs) as a box plot and violin plot for all listeners as a function of age at onset of the deafness group. A wider distribution (i.e., greater thickness) on a violin plot corresponds to a greater number of listeners with a given JND on the y-axis. Black dashed line indicates “could not determine” (CND) threshold for ITD JNDs. (**B**) Shows histograms of ITD JNDs, for each age group, binned according to levels of performance ranging from “good” (<200 μs) to “poor” (>1600 μs).

ILD JNDs were binned into five categories: <2.0, 2.01–5.0, 5.01–10.0, and >10.0 CUs, where <2.0 CUs was considered “good” and >10.0 CUs was considered very poor. “Unmeasurable” JNDs were identified as those that were >19.0 CUs. ITD JNDs were binned into five categories that also ranged from “good” to “very poor”: <200, 201–400, 401–800, 801–1600, and >1600 μs. Figure 5 shows violin plots (A) and histograms of JNDs (B) for ILDs. Figure 6 shows the corresponding data for ITD JNDs.

A Chi-square test of independence was conducted on the distributions of JNDs to determine whether the observed frequencies, according to the defined bins, differed between the childhood and adulthood groups. Distributions based on the aforementioned bins are shown using a histogram for each electrode pair location (apex, middle, base) in Figure 5B and Figure 6B for ILDs and ITD JNDs, respectively. For the ILD data set, the observed frequencies between groups and across all bins were not significantly different for any of the electrode pair locations (apex: $X^{2}$(3) = 3.25, *p* = 0.36; mid: $X^{2}$(3) = 4.81, *p* = 0.19; base: $X^{2}$(3) = 4.91, *p* = 0.18). There was only one instance in which a JND was considered “unmeasurable” for the mid electrode location. For the ITD data set, a significant difference in observed frequencies between the two groups was found for the mid electrode pair location, but not the other two locations (apex: $X^{2}$(4) = 4.60, *p* = 0.33; mid: $X^{2}$(4) = 10.13, *p* < 0.05; base: $X^{2}$(4) = 2.27, *p* = 0.69). The histograms in Figure 6B in the panel describing the mid electrode pair reveal that the childhood-onset group had many more instances of “very poor” JNDs compared to the adulthood group, indicating poorer binaural sensitivity.

Finally, we found that there was only one case (1.1%) of unmeasurable ILD JNDs, while, 18.6% of ITD JNDs were unmeasurable. This result suggests that all bilateral CI users can access ILDs, regardless of whether they lost their hearing before or after the age of 18. Our results also suggest that listeners are more prone to losing access to ITDs than to ILDs.

### 3.4. Relationship between ILD and ITD Sensitivity

**Figure 7:**
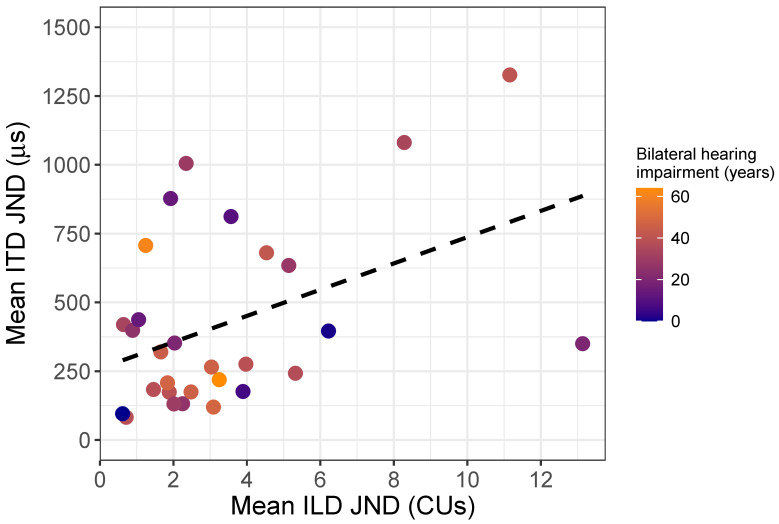
Comparison of ITD and ILD Just-noticeable differences (JNDs) as a function of years of bilateral hearing impairment. Blue-colored symbols indicate fewer years of bilateral hearing impairment and orange-colored symbols indicate greater years of bilateral hearing impairment.

Given that both ILD and ITD tasks probe the efficacy of the binaural system, we were also interested in determining if both JND measures could be best explained by matched performance across the two cues. We ran a simple linear regression using the “lm” function only on the listeners that participated in both the ILD and ITD discrimination task, using their mean JNDs. ILD JNDs were highly predictive of ITD JNDs. Thus, increased ILD JNDs corresponded to increased ITD JND (F(1,27) = 6.34, *p* < 0.05, $R^{2}$ = 0.16, regression coefficient = 47.71). Figure 7 shows the relationship between ILD and ITD JNDs (years of bilateral hearing impairment is color-coded to illustrate its effects across both measures due to its significance in interactions of ILD and ITD regression models), and the trend line illustrates the regression estimate. This finding suggests that similar mechanisms might be involved in mediating performance on binaural discrimination tasks between the two cue types. 

## 4. Discussion

Binaural hearing has become highly relevant in the clinical domain, due to the appreciation of patients’ needs to function in realistic, complex auditory environments. The research to date in patients with bilateral CIs shows a gap in performance relative to NH listeners on tasks such as sound localization, speech-in-noise understanding, and binaural sensitivity [6,13,14,56]. Patients’ clinical processors do not provide binaural cues with fidelity, contributing to weak salience of spatial locations. In addition, outcomes for bilateral CI listeners are often shaped by their acoustic and electric auditory experience, as well as changes imposed by the aging auditory system. Because spatial hearing is an important aspect of everyday listening, it is important to understand how certain etiological factors contribute to patients’ binaural sensitivity. Our study investigated the impact of factors including age, acoustic exposure/experience, and experience with electric hearing on binaural sensitivity in adults with bilateral CIs. We found that all patients had sensitivity to ILD cues for at least one place of stimulation along the cochlea. All but five patients showed sensitivity to ITD cues, even if they became deaf at a very young age. This work, which is the largest data set on adult bilateral CI patients to date, suggests that binaural sensitivity is a product of the relationship between age, acoustic, and electric experience, and we have reviewed their impact in this discussion.

### 4.1. Impact of Age-Related Factors

Prior studies have shown that early access to binaural acoustic information promotes the emergence of spatial and binaural hearing [15,16,57,58]. In children with normal hearing (NH), spatial hearing abilities and binaural sensitivity are fairly well developed by the age of four to five years [59,60,61]. Thus, the ability to achieve good spatial hearing skills depends on normal and early access to acoustic cues. In contrast, children who are deaf and hear through CIs do not have an opportunity to experience normal inputs from a young age and have poorer spatial hearing skills and reduced sensitivity to binaural cues [37,62,63,64]. Further, in children with bilateral CIs, ITD sensitivity is generally poor or non-existent when binaural inputs are disrupted in infancy, while ILD sensitivity is generally found in all children regardless of their early auditory experience. Similarly, sound localization (which requires a combination of ITDs and ILDs) is observed to some extent in all children with bilateral CIs [37,63,65,66,67,68]. These results suggest that when hearing loss occurs at a young age, sensitivity to ITDs is more difficult to achieve than sensitivity to ILDs. In those studies, the children were implanted following a relatively short period of deafness, resulting in a better-controlled method for understanding the impact of age at onset of deafness. In the present study, our participants were adults and therefore exhibited considerable heterogeneity in the amount of acoustic and electric experience they had. As a result, this heterogeneity could explain why we did not observe a strong effect of age-group for ITD sensitivity.

For age at onset of deafness, all listeners that participated in the ILD task exhibited some ILD sensitivity for at least one electrode location. In addition, we found no effect of electrode location on all measurable ILD JNDs. This is not surprising as ILDs are generally robust and are delivered reliably well through clinical processors [8,69]. When grouped categorically by age at onset of deafness, with 18 years of age as the demarcating time window, we found no differences in distributions between childhood and adult-onset of deafness for ILD JNDs at all three electrode pair locations. This finding suggests that patients with bilateral CIs can retain ILD sensitivity even if they had an early onset of deafness, and is consistent with previous reports [10,15,37].

When comparing the distributions of ITD JNDs between childhood- and adulthood-onset of deafness groups, differences were found for the mid electrode pair location but not the other locations. A critical finding from [15] was that the bilateral CI listeners who lost their hearing before the onset of spoken language (approximately at age two to three years) did not have measurable ITD JNDs. Participants who lost their hearing in mid-late childhood (between 5 and 18 years of age) and the participants with adult-onset of deafness achieved ITD sensitivity for most electrode pair locations. The present study has a larger sample size of patients and is more likely to be representative of the population of adults who have two implants. It is important to note that, when looking at the effect of electrode pair location, we found no significant effect on all measurable ITD JNDs (Figure 2B). Thus, our finding that some differences in distributions exist for ITD JNDs between childhood- and adulthood-onset of deafness groups, only partly agrees with other studies suggesting that congenital deafness has a significant negative impact on ITD sensitivity [15,35,38,58,70]. Of the 46 listeners that completed ITD testing, 41 had measurable ITD JNDs. Of the five listeners that had unmeasurable ITD JNDs, four (listeners IBJ, IBV, IBA, and IAG) were younger than 18 years of age when they lost their hearing, while only one listener (ICE) was older than 18 at the time of onset of deafness (see Table 1). Overall, the current findings lend evidence to the idea that early onset of deafness promotes the loss of ITD sensitivity, but adults with bilateral CIs who lose their hearing as adults generally have the ability to attain, or retain, sensitivity to ITDs.

Some key work in animals also supports the notion that age at onset of deafness matters for binaural sensitivity, particularly ITD sensitivity. Prior work from recordings in the inferior colliculus of rabbits has shown that early onset of deafness has a permanent effect on ITD coding because it can reduce the number of ITD-sensitive neurons and reduce their temporal coding (i.e., sensitivity to changes in pulse rates). A later onset of deafness, with a prolonged period of deafness, could simply “blur” neural ITD tuning, suggesting ITD sensitivity is potentially recoverable with appropriate binaural experience [16,70]. Additionally, these same neurons are quite robust to ILDs when stimulated directly using electrical stimulation [71].

Taken together, both the behavioral and animal data reveal that an early onset of deafness has a greater detrimental impact on ITD than ILD sensitivity. We observed a weak effect of age at onset of deafness on ITD sensitivity. In the following sections, we discuss how heterogeneity and complex interactions with multiple etiological factors may have contributed to this result. One reason we may have observed this outcome is because of the distribution of ages in our cohort: only 14 of our listeners had an age at onset less than five years of age. Hence, recruiting a greater number of listeners in the “early-onset” age group might reveal the impact of age at onset of deafness on ITD sensitivity.

Another reason we did not observe an effect could be because age at onset of deafness in humans is often difficult to determine accurately and usually relies on self-reporting from study participants. Inaccurate reporting of age at onset of deafness may introduce additional variability that led to the difference in results between studies. Further, binaural hearing in this population is impacted by the combined effects of onset of deafness with other patient-related factors. For example, age at testing had a very high correlation with years of acoustic exposure, which was included in the ILD model but was not used as a predictor in our regression to achieve the most parsimonious model determined according to Akaike’s information criterion. Other work has suggested a relationship of binaural sensitivity with age, particularly for ITDs [18,22,72,73]. In the current study, we found that age at testing was correlated with age at onset of deafness and thus could not be included together in the same analyses. Age at testing was also highly correlated with years of acoustic exposure, which was included in the ILD model, and its impact will be discussed in the next section.

### 4.2. Impact of Acoustic and Impaired Experience/Exposure

Our investigation on the impact of age at onset of deafness revealed that most listeners have binaural sensitivity and that the influence of acoustic factors on binaural sensitivity may play a larger role. Factors relating to acoustic exposure were total years of acoustic exposure and years of bilateral hearing impairment. A rather surprising finding for ILD JNDs was that the multiple regression revealed a significant and negative contribution of total years of acoustic exposure, as well as a significant interaction of years of bilateral hearing impairment with total years of acoustic exposure, for ILD JNDs (Table 3). This relationship is counterintuitive because it suggests that greater exposure was related to worse ILD sensitivity. While this might be a possibility, it should be noted that the exact years of acoustic exposure and bilateral impairment are not easy to determine. These factors depend on the degree of hearing loss in each ear prior to implantation, and whether interventions, such as a hearing aid, was used during the years of impairment. Hence, the counterintuitive result may be due to inaccuracies in our method of determining the years of acoustic exposure and years of bilateral impairment. Alternatively, we also determined that poorer ILD sensitivity was associated with increased age at testing, which may offer a more plausible explanation for the reduction in ILD sensitivity.

We discovered a strong correlation between years of acoustic exposure and age at testing (see Table 2A). When replacing years of exposure with age at testing in the multiple regression, we observed a significant main effect of age at testing as well as a significant interaction, resulting, unsurprisingly, in an almost identical model. Thus, models in Table 3 and Table 4 are difficult to distinguish from one another. Nonetheless, poorer sensitivity with greater age seems to offer a more plausible reason for our findings. Most prior work has not revealed a reliable relationship between age and ILD sensitivity [20,22,23], but a recent study demonstrated a small effect in bilateral CI and NH listeners on the ability to utilize ILDs in a lateralization task as a function of increasing age. This difference was not retained in post-hoc, pairwise comparisons, potentially due to the small sample size [18]. Thus, the poorer ILD JNDs with increases in acoustic exposure we observed in the present study may be truly due to the effects of aging. If this is not the case, poorer ILD JNDs with increases in acoustic exposure could be due to the interaction between years of acoustic exposure and age at testing since these factors could not be explored independently.

The interaction of age at testing with years of bilateral hearing impairment (Table 4) explains a unique synergistic effect between the two factors. The impact of these factors together implies that when the age of testing and years of bilateral hearing impairment decrease together, ILD JNDs should decrease. This agrees with an intuitive assumption that younger age and shorter bilateral hearing impairment should result in the best ILD sensitivity. Conversely, the interaction in our model also indicates that, when the age of testing and years of bilateral hearing impairment increases, ILD JNDs decrease. This relationship is less intuitive. If we recall that years of acoustic exposure was highly correlated with age at testing, we might begin to suspect that the reason for some listeners with older testing age having better ILD JNDs could be due to the older listeners having more years of acoustic exposure.

For ITD sensitivity, we found a significant interaction between years of bilateral hearing impairment and years with at least one CI (Table 5). This relationship suggests that, with decreases in bilateral hearing impairment and increases in electric hearing exposure (and vice versa), ITD sensitivity improves. Presumably, this is because prolonged bilateral impairment leads to poorer ITD sensitivity, and prolonged use of at least one CI leads to better ITD sensitivity. The combined evidence across studies reviewed here and the current data suggest that the interactions observed with bilateral hearing impairment on both ILD and ITD JNDs serve a complex and vital role in predicting sensitivity to binaural cues. This effect may not have been observed if either factor was explored in isolation, as in many previous studies. The implications are that adult patients with bilateral CIs benefit from having fewer years of bilateral hearing impairment to retain binaural function, and that younger listeners may show greater ILD sensitivity.

### 4.3. Impact of Electric Hearing

The third group of factors investigated was the experience with electric hearing, including years with at least one CI and years with bilateral CIs. Prior work has demonstrated the importance of experience with electric hearing for communication and everyday functioning in patients with CIs [74,75]. In the present study, the multiple regression results did not reveal strong predictions for ILD JNDs based on experience with electric hearing (Table 3 and Table 4). For the ITD data set, we found that years with at least one CI contributed to the interaction with years of bilateral hearing impairment (Table 5). It is well documented that early, simultaneous implantation in children can restore symmetric cortical organization and speech understanding [31,34,75,76,77]. Our results suggest that CI experience in adults, even if some of that experience is with one CI, may counteract the negative impact of prolonged binaural impairment on ITD processing. Conversely, other evidence suggests that CI experience does not always allow patients to fully overcome deafness-related binaural processing deficits, particularly for ITD processing [38,78]. For ILD sensitivity, experience with electric hearing does not predict performance and this is likely due to the fact that some ILD cues are still available via clinical processors [5,8,69].

While we found that CI experience appears to play a role in ITD sensitivity, it is unclear how the ITD JNDs collected with carefully-controlled stimuli presented through synchronized research processors relate to ITD performance with unsynchronized clinical processors. It is likely that the data presented here represent the “best case” scenario for binaural acuity with CIs. Hence, in order to maximize ITD/ILD sensitivity in bilateral CI users, synchronized processing combined with a new sound coding strategy, such as those proposed in other work in our laboratory [40,79], will likely be needed.

### 4.4. Relationship between Binaural Cue Types

A striking finding in our data set was that sensitivity to ILDs and ITDs was correlated. Thus far we have discussed why ITD and ILD coding might differ. In this final analysis, we found that behavioral performance on ILD and ITD sensitivity measures are related. This finding falls in line with other studies where both ILD and ITD JNDs were predictive of one another [61,80]. Spencer et al. found that the effect of the relationship between ILD and ITD JNDs was greater in NH listeners than in hearing-impaired (HI) individuals [80]. An additional aspect that was explored in that study was training. They found that mean thresholds differed from the first day of testing versus the last, for both populations, suggesting that listeners’ ability to use ITDs and ILDs appears to be plastic. While we did not directly explore the impact of training in this study, the finding in Spencer et al. [80] supports how JNDs have the potential to be plastic in HI individuals, even over a few days of testing, and could explain why we observed matched performance across the two measures of binaural sensitivity in our listeners. That is, as listeners gain additional experience using reliable ILDs and ITDs, their sensitivity may increase for one or both cues.

## 5. Conclusions and Limitations

In the present study, sensitivity to ITDs and ILDs were measured behaviorally in adult CI patients using bilaterally synchronized research processors. This study aimed to uncover how age-related, acoustic, and electric experience impact binaural sensitivity in adults with bilateral CIs. We determined that between the childhood- and adulthood-onset of deafness groups, and across a spectrum of performance from “good” to “unmeasurable” thresholds, differences in the distributions of ITD JNDs were found for only one electrode pair location. While this might suggest that the two groups differ, it is important to keep in mind that we found no effect of electrode location in the mixed-effects model, suggesting that factors other than the age at onset of deafness play a more important role in binaural sensitivity. We found that as years of acoustic exposure increased, ILD JND thresholds increased (Table 3), contrary to prior expectations. However, a strong association with age at testing (Table 2A) implies that ILD JNDs may increase with age at testing, or the interaction between age at testing and years of acoustic exposure. For ITD JNDs, we observed a moderating relationship between years of bilateral hearing impairment and years with at least one CI (Table 5). For both data sets, the years of bilateral hearing impairment appeared to play a role in the interpretation of JNDs. Finally, we found that ITD JNDs were predictive of ILD JNDs, suggesting that the ability to discriminate both binaural cues is related to one another. These results highlight the role of bilateral hearing impairment for ILDs and ITDs, further showing that ILDs may be affected by age at testing and ITDs may be affected by CI experience. More importantly, we have learned that the impact of etiological factors on binaural sensitivity is complex and is not based on one factor alone.

## Figures and Tables

**Table 1:** Listener demographics and pitch-matched electrode pairs.

Listener ID	Apex-EL	Apex-ER	Mid-EL	Mid-ER	Base-EL	Base-ER	Etiology	Age at Onset of Deafness	Age at Testing
IAG	18	22	12	18	4	10	Congenital	2	56
IAJ	19	21	14	14	6	8	Unknown; progressive since early childhood	5	65
IAZ	20	20	13	14	4	4	Hereditary	55	76
IBA	20	16	12	14	8	8	Unknown; progressive since birth	0	75
IBB	20	22	12	15	4	4	Unknown	23	44
IBD	22	20	12	12	4	4	Meniere’s; hereditary	36	81
IBF	20	22	12	13	4	6	Hereditary	38	59
IBJ	21	20	12	13	4	4	Unknown	8	26
IBK	18	22	14	13	6	6	Hereditary; noise exposure	53	70
IBL	20	20	12	12	4	4	Unknown	12	64
IBM	20	20	12	12	4	4	Hereditary	30	56
IBN	18	18	12	16	4	8	Unknown; progressive since childhood	0	65
IBO	--	--	12	12	--	--	Otosclerosis first followed by sudden bilateral loss	20	46
IBP	20	19	12	15	4	4	Meningitis in adulthood	54	61
IBQ	20	11	14	7	8	1	Meniere’s Disease-left ear Unknown-right ear	44	80
IBR	18	17	12	12	--	--	Unknown	28	57
IBU	20	20	12	12	6	6	Hereditary	20	56
IBV	20	19	12	10	4	5	Unknown illness which caused ringing in both ears	16	69
IBW	22	20	14	14	6	6	Ototoxic medication	13	56
IBX	20	22	12	13	4	4	Suspected ototoxic medication	40	70
IBY	19	17	12	12	4	7	Unknown	41	48
IBZ	18	16	12	12	4	4	Unknown; sudden loss	30	44
ICA	20	20	14	14	3	4	Childhood illnesses	13	53
ICB	18	19	12	12	4	4	Heredity	9	61
ICC	20	20	12	14	4	5	Unknown; progressive since birth	2	66
ICD	20	18	12	10	4	2	Enlarged vestibular aqueduct	3	54
ICE	20	21	12	14	4	6	Unknown	66	72
ICF	20	16	12	10	4	8	Otosclerosis	21	70
ICG	20	18	12	10	6	8	Unknown; progressive since birth	2	50
ICH	20	20	12	14	4	4	Enlarged vestibular aqueduct	2	32
ICI	18	18	12	16	4	6	Unknown	31	54
ICJ	20	16	12	12	4	6	Unknown	13	63
ICK	20	20	12	13	6	9	Noise exposure	30	69
ICL	20	19	12	18	4	6	German Measles	3	45
ICM	20	20	12	14	4	5	Unknown	20	59
ICN	16	17	12	14	8	12	Unknown; progressive since birth	4	40
ICO	18	18	12	12	4	4	Unknown; progressive since early childhood	4	32
ICP	20	22	12	14	4	10	Unknown; progressive since early childhood	3	50
ICQ	18	20	12	13	4	8	Meningitis in childhood	4	19
ICR	18	20	12	14	4	4	Radiation	27	59
ICS	18	19	12	12	4	5	Unknown	68	85
ICT	20	20	12	12	4	5	Car wreck; traumatic injury	18	20
ICV	20	20	12	14	4	7	Unknown; progressive since childhood	7	58
ICW	20	19	12	9	4	2	Congenital	0	21
ICX	18	15	12	10	8	9	Progressive since childhood; Meniere’s in adulthood	0	74

**Table 2:** (**A**) Pearson correlation coefficients for ILD JND dataset. Correlations with bold text and asterisks represent significant correlations. (**B**) Pearson correlation coefficients for the ITD JND dataset. Correlations with asterisks represent significant correlations.

**(A) Multicollinearity of the ILD JND Dataset**			
**Row**	**Column**	**Correlation Coefficient**	***p*-Value**
**Years of acoustic exposure**	**Age at testing**	**0.98**	<0.05 *
Years of acoustic exposure	Years with one CI	−0.14	0.45
Age at testing	Years with one CI	−0.06	0.74
Years of acoustic exposure	Years with bilateral CIs	0.18	0.34
Age at testing	Years with bilateral CIs	0.35	0.05
Years with one CI	Years with bilateral CIs	0.35	0.05
**Years of acoustic exposure**	**Age at Onset**	**0.56**	<0.05 *
**Age at Testing**	**Age at Onset**	**0.54**	<0.05 *
Years with one CI	Age at Onset	−0.16	0.39
Years with bilateral CIs	Age at Onset	0.08	0.65
Years of acoustic exposure	Years with bilateral hearing impairment	0.27	0.14
Age at testing	Years with bilateral hearing impairment	0.28	0.12
Years with one CI	Years with bilateral hearing impairment	0.06	0.73
Years with bilateral CIs	Years with bilateral hearing impairment	0.08	0.67
**Age at Onset**	**Years with bilateral hearing impairment**	**−0.64**	<0.05 *
**(B) Multicollinearity of ITD JND Dataset**			
**Row**	**Column**	**Correlation Coefficient**	***p*-Value**
**Years of acoustic exposure**	**Age at testing**	**0.98**	<0.05 *
Years of acoustic exposure	Years with one CI	−0.13	0.42
Age at testing	Years with one CI	−0.05	0.75
Years of acoustic exposure	Years with bilateral CIs	0.13	0.40
Age at testing	Years with bilateral CIs	0.30	0.05
**Years with one CI**	**Years with bilateral CIs**	**0.39**	<0.05 *
**Years of acoustic exposure**	**Age at Onset**	**0.53**	<0.05 *
**Age at Testing**	**Age at Onset**	**0.51**	<0.05 *
Years with one CI	Age at Onset	−0.12	0.44
Years with bilateral CIs	Age at Onset	0.05	0.75
**Years of acoustic exposure**	**Years with bilateral hearing impairment**	**0.35**	<0.05*
**Age at testing**	**Years with bilateral hearing impairment**	**0.36**	<0.05*
Years with one CI	Years with bilateral hearing impairment	0.02	0.89
Years with bilateral CIs	Years with bilateral hearing impairment	0.08	0.62
**Age at Onset**	**Years with bilateral hearing impairment**	**−0.61**	<0.05 *

**Table 3:** Model coefficients and *p*-values for ILD JND model. Asterisks denote significant predictors.

	Model Coefficients	Std. Error	*t*-Value	*p*-Value	VIF
**(Intercept)**	4.24	0.56	7.59	<0.001 *	
**Years with one CI**	−0.15	0.11	−1.41	0.17	1.04
**Years with bilateral hearing impairment**	−0.04	0.04	−1.13	0.27	1.21
**Years of acoustic exposure**	0.14	0.05	2.96	<0.05 *	1.89
**Years with bilateral hearing impairment: years of acoustic exposure**	−0.006	<0.05	−2.60	<0.05 *	1.72

**Table 4:** Model coefficients and *p*-values for the second ILD JND model. Asterisks denote the significant predictors.

	Model Coefficients	Std. Error	*t*-Value	*p*-Value	VIF
**(Intercept)**	4.28	0.56	7.67	<0.001 *	
**Years with one CI**	−0.20	0.11	−1.86	0.07	1.01
**Years with bilateral hearing impairment**	−0.04	0.03	−1.16	0.26	1.20
**Age at testing**	0.14	0.04	3.06	<0.05 *	1.87
**Age at testing: years with bilateral hearing impairment**	<−0.05	<0.001	−2.64	<0.05 *	1.72

**Table 5:** Model coefficients and p-values for ITD JND model. Asterisks denote the significant predictors.

	Model Coefficients	Std. Error	*t*-Value	*p*-Value	VIF
**(Intercept)**	429.29	46.79	9.17	<0.001 *	
**Years with bilateral hearing impairment**	−3.37	2.88	−1.12	0.25	1.01
**Years with one CI**	11.10	10.30	1.10	0.29	1.00
**Years with bilateral hearing impairment: years with one CI**	−1.90	0.72	−2.63	<0.05 *	1.01
